# A clinical guidance tool to improve the care of children hospitalized with severe pneumonia in Lusaka, Zambia

**DOI:** 10.1186/s12887-016-0665-z

**Published:** 2016-08-20

**Authors:** Catherine G. Sutcliffe, Donald M. Thea, Philip Seidenberg, James Chipeta, Lawrence Mwyanayanda, Somwe Wa Somwe, Julie Duncan, Magdalene Mwale, Justin Mulindwa, Musaku Mwenechenya, Rasa Izadnegahdar, William J. Moss

**Affiliations:** 1 Department of Epidemiology, Bloomberg School of Public Health, Johns Hopkins University, 615 North Wolfe Street, Baltimore, MD USA; 2 Center for Global Health and Development, Boston University School of Public Health, Boston University, Boston, MA USA; 3 Department of Emergency Medicine, University of New Mexico School of Medicine, Albuquerque, NM USA; 4 Department of Paediatrics, University of Zambia, School of Medicine, Lusaka, Zambia; 5 Zambia Center for Applied Health Research and Development, Lusaka, Zambia; 6 University of Missouri School of Medicine, Columbia Missouri, MO USA; 7 Bill & Melinda Gates Foundation, Seattle, WA USA

**Keywords:** Pneumonia, Sub-Saharan Africa, Global health, HIV

## Abstract

**Background:**

Pneumonia is the leading infectious cause of death among children, with approximately half of deaths attributable to pneumonia occurring in limited health resource settings of sub-Saharan Africa. Clinical guidance tools and checklists have been used to improve health outcomes and standardize care. This study was conducted to evaluate the impact of a clinical guidance tool designed to improve outcomes for children hospitalized with severe pneumonia in Zambia.

**Methods:**

This study was conducted at University Teaching Hospital in Lusaka, Zambia from October 10, 2011 to March 21, 2014 among children 1 month to 5 years of age with severe pneumonia. In March 2013, a clinical guidance tool was implemented to standardize and improve care. In-hospital mortality pre-and post-implementation was compared.

**Results:**

Four hundred forty-three children were enrolled in the pre-intervention period and 250 in the post-intervention period. Overall, 18.2 % of children died during hospitalization, with 44 % of deaths occurring within the first 24 h after admission. Mortality was associated with HIV infection status, pneumonia severity, and weight-for-height z-score. Despite improving and standardizing the care received, the clinical guidance tool did not significantly reduce mortality (relative risk: 0.89; 95 % CI: 0.65, 1.23). The tool appeared to be more effective among HIV-exposed but uninfected children and children younger than 6 months of age.

**Conclusions:**

Simple tools are needed to ensure that children hospitalized with pneumonia receive the best possible care in accordance with recommended guidelines. The clinical guidance tool was well-accepted and easy to use and succeeded in standardizing and improving care. Further research is needed to determine if similar interventions can improve treatment outcomes and should be implemented on a larger scale.

**Electronic supplementary material:**

The online version of this article (doi:10.1186/s12887-016-0665-z) contains supplementary material, which is available to authorized users.

## Background

Pneumonia is the leading infectious cause of death in children and was estimated to cause 15 % of the 6.3 million deaths globally in children younger than 5 years of age in 2013 [1]. Approximately half of deaths attributable to pneumonia occur in sub-Saharan Africa [1], where resources in the health sector are limited. Sub-Saharan Africa is also the region most affected by human immunodeficiency virus (HIV) infection and is home to over 90 % of the 2.6 million children infected with HIV globally [2]. HIV-infected children are at increased risk of severe pneumonia and poor outcomes, including death [3–6]. Increasing evidence suggests that HIV-exposed but uninfected children may also be at increased risk and this group is increasing as successful programs to prevent mother-to-children transmission of HIV expand [5, 7].

Guidelines on the management of children hospitalized with severe pneumonia provide recommendations for diagnosis, treatment and supportive care [8]. In addition to antibiotic therapy, children should receive oxygen therapy if oxygen saturation is less than 90 % as well as supportive care, including removal of secretions, treatment for fever or wheeze, and receipt of maintenance fluids and nutritional support. HIV-exposed infants should receive cotrimoxazole for presumptive pneumocystis pneumonia [9]. Addressing these needs requires not only adequate supplies of antibiotics, oxygen, bronchodilators and appropriate equipment, but frequent monitoring by nurses and physicians to identify and address each child’s medical needs. In resource-limited settings, many of these elements may be challenging to deliver. Determining the need for oxygen can be difficult given the absence or limited availability of pulse oximeters in health facilities. When oxygen is needed, shortages are common, and when available the equipment required for administration and monitoring may not be present or functional [10]. Critical shortages of healthcare workers are also common throughout the region, with only 2.3 health workers estimated to be available per 1000 population [11]. Consequently, many children with pneumonia may not receive the recommended standard of care and may experience poorer outcomes.

Clinical guidance tools and checklists have been used in critical care, anesthesia and surgical settings with demonstrated improvement in patient safety and outcomes [12–15], as well as increased adherence to published guidelines leading to standardized patient care [16]. This study was conducted to evaluate the impact of a clinical guidance tool designed to improve outcomes for children hospitalized with severe pneumonia in Lusaka, Zambia.

## Methods

### Setting and population

This study was conducted at the University Teaching Hospital (UTH) in Lusaka, Zambia within a sample of children enrolled into the Pneumonia Etiology Research for Child Health (PERCH) study [17]. Zambia is categorized as a lower middle income country with 60.5 % of the population living below the poverty level [18]. In 2013, the maternal and under-5 mortality rates were estimated to be 398 per 100,000 live births and 75 per 1000 live births, respectively [19]. Immunization coverage with the third dose of diphtheria-pertussis-tetanus was estimated to be 79 % [18]. *Haemophilus influenzae* type b and pneumococcal conjugate vaccines were introduced into the national immunization program in 2004 and 2013, respectively [20]. In 2013, the prevalence of HIV among adults was estimated to be 13.3 % in Zambia and 16.3 % in Lusaka [19].

UTH is a 1500-bed academic center and tertiary care facility with 447 pediatric beds. UTH serves as a referral hospital for approximately 1.3 million people living within the greater Lusaka District. The Department of Paediatrics and Child Health operates a 24-h outpatient (OPD) and in-patient clinical care service with laboratory, radiological and nutritional support care services. The Department of Paediatrics and Child Health consists of an admission ward, 3 general pediatric wards, a malnutrition ward, pediatric haemato-oncology wards, an isolation infectious disease ward, a pediatric intensive care unit and a neonatal intensive care unit. All children are first evaluated in the OPD. Severely ill children, including the majority of children with pneumonia, are triaged to the emergency room for resuscitation and stabilization, including administration of intravenous fluids for correction of shock or dehydration, oxygen therapy and empirical antibiotic therapy. Basic diagnostic investigations such as complete blood counts, biochemistry tests (liver and renal function tests, blood sugar, etc.), microbiology procedures (gram stains, sputum for acid fast bacilli and blood cultures) and chest radiographs are commonly performed, when available, on children admitted with pneumonia. Since 2005, routine opt out HIV testing for every admitted child has been performed [21].

### PERCH Study

This study was nested within the PERCH study site in Zambia. The design and methods of the PERCH study were described elsewhere [17, 22, 23]. Briefly, PERCH is a multi-center, case–control study to determine the etiology of pneumonia among children younger than 5 years of age living in developing countries. All children admitted to UTH between the ages of 28 days and 59 months with severe or very severe pneumonia, consistent with the World Health Organization definitions when the study began [24], and living within a pre-defined area of Lusaka were eligible for enrollment. Children were excluded from the study if they were hospitalized in the 14 days prior to admission or if they had previously been enrolled 30 days prior to admission. Written informed consent for participation was obtained from parents or guardians. The PERCH study was conducted from October 2011 to March 2014.

Suspected cases of severe or very severe pneumonia presenting to UTH were assessed for eligibility using a pre-screening assessment tool. Upon enrollment into the PERCH study, an extensive demographic and clinical history was obtained from the caregiver and the child underwent a clinical assessment, including chest radiography. Specimens collected included induced sputum, blood, pleural fluid (if appropriate), nasal and throat swabs, and urine. At 24 and 48 h following admission, all children underwent a limited clinical assessment, including measurement of respiratory rate, and oxygen saturation, and evaluation for the presence of wheeze and lower chest wall indrawing. At hospital discharge, oxygen saturation, respiratory status and wheeze were assessed and the discharge diagnosis and clinical status of the child were recorded. Aside from these study assessments, clinical care was provided by UTH nursing and physician clinical ward staff, neither of which were part of the PERCH study team.

HIV testing of children was initially performed upon admission by hospital staff according to the opt-out HIV testing protocol. In February 2012, the PERCH protocol was changed to include rapid HIV testing (Abbott Determine®, Abbott Park, IL) of both children and consenting mothers at enrollment. Children who tested positive underwent confirmatory testing according to national guidelines (HIV DNA-PCR testing for children younger than 18 months of age and detection of plasma antibodies by enzyme immunoassay [Organon Tecknika, Boxtel, The Netherlands] for children 18 months of age and older). Children of HIV-infected mothers were given cotrimoxazole according to national guidelines.

### Study procedures

In 2012, investigators observed that the PERCH site in Zambia had a higher case-fatality ratio among participants than other sites and hypothesized that this may be in part due to a lack of standardization in clinical care. To standardize and improve the care of study children provided by the ward staff, a clinical guidance tool was developed in collaboration with the clinical care team from the Department of Paediatrics and Child Health. The tool was based on the WHO recommendations for the management of acute respiratory illness at a referral health facility and included best practices specific to HIV-infected and exposed children (Table 1; Additional file 1). To standardize antibiotic regimens provided to children with pneumonia, 4 preferred regimens were listed. To ensure that HIV exposure and infection status were appropriately ascertained and managed, prompts were provided to the clinical ward staff to obtain and follow-up on results for HIV testing as well as referral and administration of cotrimoxazole. Prompts to the ward staff to assess respiratory rate, temperature and oxygen saturation were included every 6 hours to ensure the child was appropriately monitored. Additional prompts were provided to assess the nutritional status of the child with referral for nutritional supplements as necessary. With approval by the Department of Paediatrics and Child Health, the clinical guidance tool was implemented on March 4, 2013 for study participants. Clinical decisions regarding management and treatment were made by the ward clinical staff but the tool was available at the child’s bedside.

**Table 1:** Components of the clinical guidance tool

On admission	- Antibiotics administered (4 regimens recommended) - Assessment of HIV-exposure with recommendations for HIV testing and cotrimoxazole prophylaxis - Assessment of nutritional status with documentation of feeding method implemented - Documentation of assessments performed, including blood culture, complete blood count and chest x-ray - Assessment of oxygen status with recommendations for supplemental oxygen - Assessment of the need for supportive care (shock, fever, wheezing, nasal secretions, maintenance fluids) with recommendations provided
At 48 h	- Assessment of criteria for treatment failure with recommendations for switching antibiotic regimens
Ongoing care	Assessment every 6 hours of: - Room air saturation - Chest indrawing - Respiratory rate
Discharge	- Assessment of criteria for discharge - Documentation of oral regimen prescribed - Documentation of discharge instructions provided to the caregiver - Assessment of HIV-exposure status with recommendations for follow-up - Assessment of nutritional status with recommendations for follow-up

Study nurses were employed on both day and night shifts and were responsible for completing the tool based on information in the child’s medical chart. Study staff assessed treatment failure at 24 and 48 h after admission and the clinical status of the child upon discharge. Study staff collected data on adherence to the assessments described in the tool. Study nurses ensured that medications and fluids were administered as prescribed, oxygen therapy continued when appropriate, and vital signs measured as recommended. In addition, they assisted ward staff in suctioning the airways of study participants. Ward nurses and physicians were alerted to deviations from the tool within hours of determination so remedies could be enacted. During the intervention period, oxygen was more readily available at the hospital as well as certain antibiotics (e.g. intravenous cotrimoxazole).

This study was approved by the Boston University, Johns Hopkins Bloomberg School of Public Health, and Zambian ERES Converge Institutional Review Boards.

### Statistical analysis

All children enrolled in the PERCH study were included in this analysis, which consisted of a pre-post study design comparing clinical outcomes, including duration of hospital stay and mortality, before and after implementation of the clinical guidance tool. Children enrolled from October 10, 2011 to March 3, 2013 were included in the pre-intervention group and children enrolled from March 4, 2013 to March 21, 2014 were included in the post-intervention group. HIV infection status was defined based on maternal history, HIV antibody testing and HIV DNA testing. Children were classified as HIV infected if they had previous documentation of HIV infection, were currently receiving antiretroviral therapy or if they had a positive HIV (as per protocol and based on age) test performed as part of the study. Children were classified as exposed to HIV if they were infected with HIV, if they were HIV seropositive, if the mother was documented to have had a positive HIV test during pregnancy, if the mother was reported to be HIV seropositive during pregnancy (in the absence of a negative serological test if <7 months of age), or if the mother tested positive at enrollment and the child was younger than 7 months of age. Children older than 7 months of age with a negative reported maternal history who were HIV seronegative were presumed to be HIV-unexposed. Based on exposure and infection data, HIV status was categorized as HIV-unexposed and uninfected, HIV-exposed but uninfected, and HIV-exposed and infected. Nine children were uninfected with unknown exposure status and classified as HIV-unexposed and uninfected. Four children were HIV-exposed with unknown infection status and were classified as HIV-exposed but uninfected. Weight-for-age (WAZ) and weight-for-height (WHZ) z-scores were calculated based on the WHO growth standards to assess malnutrition and wasting, respectively [25]. High pneumonia season was defined as enrollment in January, February, March, November and December of any given year based on historical increases in admissions for respiratory infections [unpublished data].

Differences in demographic and clinical characteristics between the pre- and post-intervention groups were assessed using chi-square tests for categorical variables and Wilcoxon rank sum tests for continuous variables. Poisson regression with robust variance estimation was used to compare the risk of in-hospital mortality in the pre- and post-intervention periods. Characteristics found to differ significantly (*p* < 0.05) between groups were included in the model. As some children were missing information on WHZ at enrollment, an indicator for ‘missing’ was used for WHZ to include all children in the models. Interactions with pneumonia severity, HIV infection status, age, WHZ and season were explored.

Sensitivity analyses were conducted restricting the study population to children remaining in care for more than 24 h as they were hypothesized to receive the most clinical benefits from the tool, and to children enrolled in the study over the same calendar period in the pre- (March 2012-March 2013) and post-intervention (March 2013-March 2014) groups, with no significant difference in the results. In addition, multiple imputation methods were explored to address missing data with no significant difference in the results.

## Results

Between October 10, 2011 and March 21, 2014, 693 children were enrolled in the PERCH study, 443 in the pre-intervention period and 250 in the post-intervention period. Approximately half the children were male and younger than 6 months of age at enrollment, with no difference by intervention period (Table 2). Children enrolled in the post-intervention period were significantly more likely than those enrolled in the pre-intervention period to be admitted with very severe pneumonia (38.4 % vs. 28.7 %; *p* = 0.009), to be HIV-infected (19.2 % vs. 14.2 %; *p* = 0.02), to have a normal WHZ (70.4 % vs. 46.9 % with WHZ −2 to 2; *p* < 0.0001), and to have a normal chest x-ray (40.9 % vs. 30.1 %; *p* = 0.02).

**Table 2:** Characteristics of children hospitalized with pneumonia at the University Teaching Hospital in Lusaka, Zambia

Characteristics at study enrollment	Pre-intervention group (*N* = 443)	Post-intervention group (*N* = 250)	*p*-value
Age (months)			0.31
0–5	240 (54.2)	124 (49.6)	
6–11	101 (22.8)	64 (25.6)	
12–23	59 (13.3)	43 (17.2)	
24–59	43 (9.7)	19 (7.6)	
Male sex	234 (52.8)	133 (53.2)	0.92
Hib conjugate vaccine status			0.25
Received ≥1 dose	332 (81.0)	192 (84.6)	
Received 0 doses	78 (19.0)	35 (15.4)	
Missing	33	23	
Pneumococcal conjugate vaccine status			<0.0001
Received ≥1 dose	0	37 (18.1)	
Received 0 doses	0	168 (81.9)	
Missing	0	45	
HIV infection status			0.02
Unexposed/uninfected	273 (61.6)	162 (64.8)	
Exposed/uninfected	107 (24.1)	40 (16.0)	
Infected	63 (14.2)	48 (19.2)	
Weight for age z-score			0.35
≤ − 3	74 (16.7)	32 (12.8)	
− 3 to −2	80 (18.1)	44 (17.6)	
> − 2	288 (65.0)	174 (69.6)	
Missing	1	0	
Weight for height z-score			<0.0001
≤ − 3	51 (12.2)	14 (5.8)	
− 3 to − 2	46 (11.0)	22 (9.2)	
− 2 to 2	196 (46.9)	169 (70.4)	
> = 2	125 (29.9)	35 (14.6)	
Missing	25	10	
Mother’s education			0.7
None	20 (4.6)	10 (4.0)	
Primary	170 (39.0)	105 (42.2)	
Secondary or more	246 (56.4)	134 (53.8)	
Missing	7	1	
Very severe pneumonia	127 (28.7)	96 (38.4)	0.009
Oxygen saturation ≤92 %	191 (43.3)	113 (45.2)	0.63
Chest x-ray result			0.02
Primary endpoint pneumonia^a^	79 (20.5)	49 (22.3)	
Other infiltrates	67 (17.4)	24 (10.9)	
Primary endpoint pneumonia^a^ & other infiltrates	44 (11.4)	26 (11.8)	
Normal	117 (30.4)	90 (40.9)	
Uninterpretable	78 (20.3)	31 (14.1)	
Missing	58	30	
Admitted in high pneumonia season	192 (43.3)	96 (38.4)	0.21

^a^Images as showing alveolar consolidation or pleural effusion

**Fig. 1:**
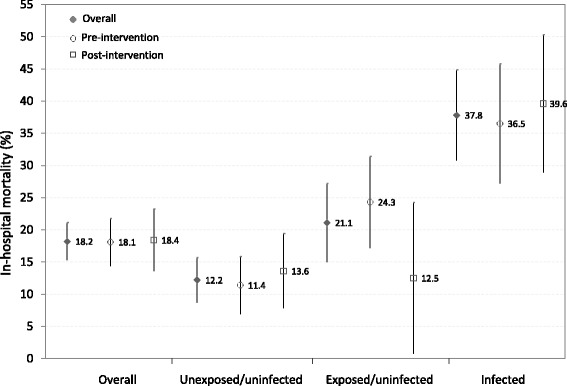
Mortality among children hospitalized with pneumonia in Zambia, by intervention and HIV infection status. Note: bars around estimates represent 95 % confidence intervals

The median duration of hospitalization did not differ between children enrolled pre- (median: 6; IQR: 4–9; range: 1–50) and post-intervention (median: 5; IQR: 3–9; range: 1–39; *p* = 0.24). Overall, 18.2 % of children died during hospitalization (Fig. 1) with 44 % of deaths occurring within the first 24 h after admission (41 % pre- and 50 % post-intervention; *p* = 0.34). Mortality among HIV-exposed but uninfected children (21.1 %) and HIV-infected children (33.3 %) was significantly higher than among HIV-unexposed and uninfected children (12.2 %; *p* < 0.0001; Fig. 1). No significant difference in mortality was observed by intervention (relative risk [RR]: 1.01; 95 % confidence interval [CI]: 0.73, 1.41; Table 3). After adjusting for pneumonia severity, HIV infection status, WHZ and pneumonia season, a small but non-significant decrease in mortality in the post-intervention period was observed (RR: 0.89; 95 % CI: 0.65, 1.23).

**Table 3:** Risk factors for in-hospital mortality among children hospitalized with severe pneumonia in Zambia

	N	In-hospital mortality (%)	Crude relative risk (95 % CI)	Adjusted relative risk (95 % CI)
Group				
Pre-intervention	443	18.1	1	1
Post-intervention	250	18.4	1.01 (0.73, 1.41)	0.89 (0.65, 1.23)
Age (months)				
0–5	364	18.7	1	
6–11	165	20.0	1.07 (0.74, 1.55)	
12–23	102	17.7	0.94 (0.59, 1.51)	
24–59	62	11.3	0.60 (0.29, 1.25)	
Pneumonia severity				
Severe	470	11.5	1	1
Very severe	223	32.3	2.81 (2.05, 3.85)	2.79 (2.04, 3.82)
HIV infection status				
Unexposed/uninfected	435	12.2	1	1
Exposed/uninfected	147	21.1	1.73 (1.16, 2.59)	1.73 (1.17, 2.56)
Infected	111	37.8	3.11 (2.19, 4.39)	2.86 (2.01, 4.08)
Weight for height z-score				
≤ −3	65	32.3	2.23 (1.45, 3.42)	1.85 (1.19, 2.86)
− 3 to − 2	68	25.0	1.72 (1.06, 2.79)	1.57 (0.97, 2.55)
− 2 to 2	365	14.5	1	1
> =2	160	13.8	0.95 (0.60, 1.50)	1.00 (0.65, 1.55)
Pneumonia season				
Low	405	21.7	1	1
High	288	13.2	0.61 (0.43, 0.86)	0.85 (0.59, 1.21)

Differences in the effect of the intervention on in-hospital mortality were observed by HIV infection status and age, with the majority of the effect observed among HIV-exposed but uninfected children (RR: 0.48; 95 % CI: 0.22, 1.04) and children younger than 6 months of age (RR: 0.64; 95 % CI: 0.40, 1.02) (Table 4). No differences were observed by pneumonia severity, season or WHZ.

**Table 4:** Differences in the effect of the intervention on mortality by age and HIV infection status

	N pre/post intervention period	In hospital mortality in the pre/post intervention period (%)	Adjusted relative risk (95 % CI)	*p*-value for interaction
Age^a^				
< 6 months	240/124	20.8/14.5	0.64 (0.40, 1.02)	0.05
≥ 6 months	203/126	14.8/22.2	1.23 (0.79, 1.92)	ref
HIV infection status^b^				
Unexposed/uninfected	273/162	11.4/13.6	1.20 (0.73, 1.99)	ref
Exposed/uninfected	107/40	24.3/12.5	0.48 (0.22, 1.04)	0.05
Infected	63/48	36.5/39.6	0.82 (0.51, 1.33)	0.28
Pneumonia severity^c^				
Severe	316/154	11.7/11.0	0.97 (0.57, 1.64)	ref
Very severe	127/96	33.9/30.2	0.85 (0.58, 1.24)	0.68
Pneumonia season^d^				
Low	251/154	21.9/21.4	0.83 (0.57, 1.20)	ref
High	192/96	13.0/13.5	1.08 (0.60, 1.93)	0.45
Weight-for-age z-score^e^				
< −2	97/36	28.9/27.8	0.58 (0.31, 1.10)	0.21
≥ −2	321/204	14.3/14.2	0.93 (0.63, 1.37)	ref

^a^model was adjusted for weight-for-age z-score, severity of pneumonia, HIV status, and pneumonia season

^b^model was adjusted for age, weight-for-age z-score, severity of pneumonia, and pneumonia season

^c^model was adjusted for weight-for-age z-score, HIV status, and pneumonia season

^d^model was adjusted for weight-for-age z-score, HIV status, and severity of pneumonia

^e^model was adjusted for age, HIV status, severity of pneumonia, and pneumonia season

To evaluate potential mechanisms mediating the effect, processes impacted by the clinical tool were evaluated. Four standard antibiotic regimens were promoted during the intervention but the proportion of children receiving other regimens did not significantly differ between the pre- and post-intervention periods (8.8 % vs. 12.1 %; *p* = 0.17). Increased monitoring of supportive care was promoted and the proportion of children with oxygen saturation ≤92 % receiving oxygen on admission (83.3 % vs. 93.8 %; *p* = 0.02) and at 48 h follow-up (76.4 % vs. 95.3 %; *p* = 0.001) increased significantly from the pre- to the post-intervention period. Management of HIV-exposed children was also promoted and a non-significant increase in the proportion of HIV-infected children receiving cotrimoxazole at admission or during follow-up was observed from the pre- to the post-intervention period (85.7 % vs. 95.8 %; *p* = 0.08). Similar increases were not observed among HIV-exposed but uninfected children (68.3 % vs. 67.4 %; *p* = 0.91).

Adherence to the tool was assessed in terms of completeness of the forms. Sixty-seven percent of items were completed at least 90 % of the time, with item completeness ranging from 67 to 100 %. Lower completeness was observed for items documenting management of a test result or measure (e.g. documenting administration of paracetamol if fever was recorded).

## Discussion

In this study of children hospitalized with severe and very severe pneumonia, 18.2 % of children died while in hospital and almost half within the first 24 h of admission. Mortality was associated with HIV exposure and infection, pneumonia severity, and severe malnutrition. Use of a clinical guidance tool improved the care children received and was associated with a small but non-significant decrease in overall in-hospital mortality.

The prevalence of HIV infection among hospitalized children with severe or very severe pneumonia in sub-Saharan Africa ranged from 45-65 % in prior studies, with a case fatality ratio of 20-34 %, 3 to 6-fold higher than among HIV-uninfected children [3, 4, 6, 7]. While the prevalence of HIV infection in this study was only 16 %, HIV-infected children were almost 3 times more likely to die in hospital than HIV-uninfected children. Similar to other studies [5], in-hospital mortality among HIV-exposed but uninfected children was lower than among HIV-infected children but almost two-fold higher than among HIV-uninfected children. Reasons for the increased risk of mortality among HIV-exposed but uninfected children are multifactorial and could include lower levels of maternally-acquired antibodies to respiratory pathogens, suboptimal breastfeeding, undernutrition, parental death, increased exposure to infectious pathogens from HIV-infected adults, and potential immunological abnormalities [26, 27].

The clinical guidance tool used in this study was developed to improve outcomes through standardization of care, with particular focus on identification and management of HIV exposure and infection and provision of supportive care. Similar to other studies [16, 28], implementation of the tool improved provision of recommended care for children exposed and infected with HIV as well as children with severe pneumonia. HIV-infected children were more likely to receive cotrimoxazole and children in need were more likely to receive oxygen. Despite recommending a limited set of antibiotics, there was no impact on the range of regimens prescribed in the post-intervention period. This may have been due to the limited availability of certain antibiotics at the pharmacy or resistance by physicians in changing their prescribing habits. In discussions with study staff, nurses believed the clinical guidance tool improved the care children received through closer and more consistent monitoring and more rapid identification and correction of problems and abnormalities. They felt that the tool was simple and easy to use, as demonstrated by the high observed adherence, and that it was well accepted by the clinical ward staff and caregivers. They all felt, however, that it would be difficult to continue using the clinical guidance tool beyond the study given the shortage of ward staff and inconsistent availability of resources at the hospital.

Despite improvements and standardization of care, only a small impact on overall in-hospital mortality was observed. This may have been due in part to the critical condition of the children at the time of hospitalization, as almost half of the deaths occurred within the first 24 h of admission. By the time these children reached the hospital, death may not have been preventable regardless of the care received. Delays in bringing a child with severe pneumonia to the hospital may result from delayed recognition of symptoms by the mother or delays due to the health system. Children are usually seen first at a health center and referred to the hospital by a healthcare worker. The health center is generally busy and children may not be seen for several hours. In addition, ambulance services may be slow or absent and further delay arrival at the hospital. Efforts to educate new mothers on the signs and symptoms of pneumonia and to improve the referral system may improve care seeking behaviors and ultimately outcomes [29].

The clinical guidance tool appeared to have the greatest impact among children younger than 6 months of age and HIV-exposed but uninfected children. Reasons for this are unclear, but these children may be most likely to achieve better outcomes with improved supportive care, nutritional support and prompt identification and management of HIV exposure. This study was not designed or powered to evaluate the effect of the intervention among subgroups and further research is needed to confirm these findings. As the largest number of pneumonia cases and deaths were among children younger than 6 months of age and the number of HIV-exposed children will continue to increase, simple, clinical tools to improve pneumonia outcomes would be useful.

There has been an increased interest in the implementation of best clinical practices, clinical guidance tools and checklist approaches for clinical care or patient harm reduction in a wide variety of healthcare settings, including low and middle income countries. Programs such as the WHO’s Safe Surgery Saves Lives [30] and Safe Childbirth Checklist [31] have been primarily developed for and implemented in low and middle income settings where the burden of disease is greatest, the perceived need for standardized evidence-based clinical guidelines to improve outcomes the strongest, and barriers to introducing costly interventions involving large numbers of healthcare providers the greatest. These efforts have been met with mixed results [32–34], including the results found in our current study. While there is great hope that simply introducing standardized evidence-based guidelines or checklists in any healthcare setting will lead to better quality of care and improved outcomes, in reality changing healthcare provider and institutional behavior, and sustaining these changes over time, is much more difficult [35, 36]. Understanding cultural and institutional barriers to change, recognizing the intricate psychology for changing human behavior, realizing the impact of politics on workplace environments, providing timely feedback to providers and monitoring efforts, and choosing appropriate and simplified interventions in the right setting for the targeted patient population all likely impact the adoption of standardized approaches and their success.

This study had several limitations. First, this study was nested within the PERCH study and was conceived and implemented in the middle of the study. Consequently, the sample size was limited and the study was not powered to detect small decreases in mortality. Second, as the tool was partly implemented by study staff, results may reflect implementation of the tool under enhanced conditions. Third, the study compared 2 periods of time pre and post-intervention. In addition to implementing the tool, other improvements were made, including increasing coverage of study staff. Improvements in mortality or outcomes may not be attributable solely to the clinical guidance tool. Lastly, children enrolled in the pre and post-intervention periods differed on several characteristics related to mortality. While adjustments were made in the statistical analysis, it is possible that unmeasured confounders remained.

## Conclusions

At a large, urban hospital in Lusaka, Zambia, mortality among children hospitalized with severe and very severe pneumonia was high. Simple tools are needed to ensure that these children receive the best possible care in accordance with recommended guidelines. The clinical guidance tool developed and evaluated in this study was well-accepted and easy to use and succeeded in standardizing and improving care. Further research is needed to determine if similar interventions can improve outcomes and should be implemented on a larger scale.

## Abbreviations

CI, confidence interval; Hib, *Haemophilus influenzae* type b; HIV, human immunodeficiency virus; IQR, interquartile range; OPD, out-patient department; PERCH, Pneumonia Etiology Research for Child Health (PERCH) study; RR, relative risk; UTH, University Teaching Hospital; WAZ, weight-for-age z-score; WHZ, weight-for-height z-score

## Additional file

Additional file 1: Clinical guidance tool implemented at the University Teaching Hospital in Lusaka, Zambia (DOCX 62 kb)

## References

[1] Liu L, Oza S, Hogan D, Perin J, Rudan I, Lawn JE, Cousens S, Mathers C, Black RE (2015). Global, regional, and national causes of child mortality in 2000–13, with projections to inform post-2015 priorities: an updated systematic analysis. Lancet.

[2] UNAIDS (2015). How AIDS changed everything. MDG 6: 15 years, 15 lessons of hope from the AIDS response.

[3] Graham SM, Mtitimila EI, Kamanga HS, Walsh AL, Hart CA, Molyneux ME (2000). Clinical presentation and outcome of Pneumocystis carinii pneumonia in Malawian children. Lancet.

[4] Graham SM (2003). Impact of HIV on childhood respiratory illness: differences between developing and developed countries. Pediatr Pulmonol.

[5] Gray D, Zar HJ (2009). Management of community-acquired pneumonia in HIV-infected children. Expert Rev Anti Infect Ther.

[6] Jeena PM (2008). Can the burden of pneumonia among HIV-infected children be reduced?. Bull World Health Organ.

[7] McNally LM, Jeena PM, Gajee K, Thula SA, Sturm AW, Cassol S, Tomkins AM, Coovadia HM, Goldblatt D (2007). Effect of age, polymicrobial disease, and maternal HIV status on treatment response and cause of severe pneumonia in South African children: a prospective descriptive study. Lancet.

[8] WHO (2013). Pocket Book of Hospital Care for Children. Guidelines for the Management of Common Childhood Illnesses.

[9] WHO (2010). WHO recommendations on the management of diarrhoea and pneumonia in HIV-infected infants and children.

[10] Enarson P, La Vincente S, Gie R, Maganga E, Chokani C (2008). Implementation of an oxygen concentrator system in district hospital paediatric wards throughout Malawi. Bull World Health Organ.

[11] WHO (2006). Working together for health-The World Health Report 2006.

[12] Pronovost P, Needham D, Berenholtz S, Sinopoli D, Chu H, Cosgrove S, Sexton B, Hyzy R, Welsh R, Roth G (2006). An intervention to decrease catheter-related bloodstream infections in the ICU. N Engl J Med.

[13] Runciman WB, Morris RW, Watterson LM, Williamson JA, Paix AD (2005). Crisis management during anaesthesia: cardiac arrest. Qual Saf Health Care.

[14] Haynes AB, Weiser TG, Berry WR, Lipsitz SR, Breizat AH, Dellinger EP, Herbosa T, Joseph S, Kibatala PL, Lapitan MC (2009). A surgical safety checklist to reduce morbidity and mortality in a global population. N Engl J Med.

[15] Weiser TG, Berry WR (2013). Review article: perioperative checklist methodologies. Can J Anaesth.

[16] Berenholtz SM, Pronovost PJ, Lipsett PA, Hobson D, Earsing K, Farley JE, Milanovich S, Garrett-Mayer E, Winters BD, Rubin HR (2004). Eliminating catheter-related bloodstream infections in the intensive care unit. Crit Care Med.

[17] Levine OS, O’Brien KL, Deloria-Knoll M, Murdoch DR, Feikin DR, DeLuca AN, Driscoll AJ, Baggett HC, Brooks WA, Howie SR (2012). The Pneumonia Etiology Research for Child Health Project: a 21st century childhood pneumonia etiology study. Clin Infect Dis.

[18] The World Bank [http://www.worldbank.org/]. Accessed 17 Mar 2015.

[19] Central Statistical Office (CSO) [Zambia] MoHMZ, and ICF International (2014). Zambia Demographic and Health Survey 2013–14.

[20] IVAC (2015). VIMS Report: Global Vaccine Introduction. January 2015.

[21] Kankasa C, Carter RJ, Briggs N, Bulterys M, Chama E, Cooper ER, Costa C, Spielman E, Katepa-Bwalya M, M’Soka T (2009). Routine offering of HIV testing to hospitalized pediatric patients at university teaching hospital, Lusaka, Zambia: acceptability and feasibility. J Acquir Immune Defic Syndr.

[22] Scott JA, Wonodi C, Moisi JC, Deloria-Knoll M, DeLuca AN, Karron RA, Bhat N, Murdoch DR, Crawley J, Levine OS (2012). The definition of pneumonia, the assessment of severity, and clinical standardization in the Pneumonia Etiology Research for Child Health study. Clin Infect Dis.

[23] Deloria-Knoll M, Feikin DR, Scott JA, O’Brien KL, DeLuca AN, Driscoll AJ, Levine OS (2012). Identification and selection of cases and controls in the Pneumonia Etiology Research for Child Health project. Clin Infect Dis.

[24] WHO (1991). Technical basis for the WHO recommendations on the management of pneumonia in children at first-level health facilities.

[25] The WHO child growth standards [http://www.who.int/childgrowth/en/index.html]. Accessed 1 Mar 2014.

[26] Filteau S (2009). The HIV-exposed, uninfected African child. Trop Med Int Health.

[27] Gesner M, Papaevangelou V, Kim M, Chen SH, Moore T, Krasinski K, Borkowsky W (1994). Alteration in the proportion of CD4 T lymphocytes in a subgroup of human immunodeficiency virus-exposed-uninfected children. Pediatrics.

[28] Mendu ML, Schneider LI, Aizer AA, Singh K, Leaf DE, Lee TH, Waikar SS (2014). Implementation of a CKD checklist for primary care providers. Clin J Am Soc Nephrol.

[29] Geldsetzer P, Williams TC, Kirolos A, Mitchell S, Ratcliffe LA, Kohli-Lynch MK, Bischoff EJ, Cameron S, Campbell H (2014). The recognition of and care seeking behaviour for childhood illness in developing countries: a systematic review. PLoS ONE.

[30] World Alliance for Patient Safety/World Health Organization. Safe Surgery Saves Lives. Geneva, Switzerland: WHO; 2008.

[31] WHO Safe Childbirth Checklist [http://www.who.int/patientsafety/implementation/checklists/childbirth-checklist/en/]. Accessed 15 Feb 2016.

[32] Andrews B, Muchemwa L, Kelly P, Lakhi S, Heimburger DC, Bernard GR (2014). Simplified severe sepsis protocol: a randomized controlled trial of modified early goal-directed therapy in Zambia. Crit Care Med.

[33] Jacob ST, Banura P, Baeten JM, Moore CC, Meya D, Nakiyingi L, Burke R, Horton CL, Iga B, Wald A (2012). The impact of early monitored management on survival in hospitalized adult Ugandan patients with severe sepsis: a prospective intervention study. Crit Care Med.

[34] Spector JM, Agrawal P, Kodkany B, Lipsitz S, Lashoher A, Dziekan G, Bahl R, Merialdi M, Mathai M, Lemer C (2012). Improving quality of care for maternal and newborn health: prospective pilot study of the WHO safe childbirth checklist program. PLoS ONE.

[35] Bosk CL, Dixon-Woods M, Goeschel CA, Pronovost PJ (2009). Reality check for checklists. Lancet.

[36] Grol R, Grimshaw J (2003). From best evidence to best practice: effective implementation of change in patients’ care. Lancet.

