# Erratum to: A clinical guidance tool to improve the care of children hospitalized with severe pneumonia in Lusaka, Zambia

**DOI:** 10.1186/s12887-016-0698-3

**Published:** 2016-09-29

**Authors:** Catherine G. Sutcliffe, Donald M. Thea, Philip Seidenberg, James Chipeta, Lawrence Mwananyanda, Somwe Wa Somwe, Julie Duncan, Magdalene Mwale, Justin Mulindwa, Musaku Mwenechenya, Rasa Izadnegahdar, William J. Moss

**Affiliations:** 1 Department of Epidemiology, Bloomberg School of Public Health, Johns Hopkins University, 615 North Wolfe Street, Baltimore, MD USA; 2 Center for Global Health and Development, Boston University School of Public Health, Boston University, Boston, MA USA; 3 Department of Emergency Medicine, University of New Mexico School of Medicine, Albuquerque, NM USA; 4 Department of Paediatrics, University of Zambia, School of Medicine, Lusaka, Zambia; 5 Zambia Center for Applied Health Research and Development, Lusaka, Zambia; 6 University of Missouri School of Medicine, Columbia Missouri, MO USA; 7 Bill & Melinda Gates Foundation, Seattle, WA USA

## Erratum

After publication of the original article [1] it was brought to our attention that author Lawrence Mwananyanda was incorrectly included as Lawrence Mwyanayanda. The correct spelling of the name is included in the author list of this erratum and has also been updated in the original article.
