# Supplementary material for: A clinical guidance tool to improve the care of children hospitalized with severe pneumonia in Lusaka, Zambia
Source: BMC Pediatr. 2016 Aug 20;16:136. doi: 10.1186/s12887-016-0665-z (PMC4992255; doi:10.1186/s12887-016-0665-z)
Supplement: Additional file 1: — Clinical guidance tool implemented at the University Teaching Hospital in Lusaka, Zambia (DOCX 62 kb) [file 12887_2016_665_MOESM1_ESM.docx]

|  | **ON ADMISSION** | | | **Ward number:** |
| --- | --- | --- | --- | --- |
| **ANTIBIOTICS**  **Initial therapy (choose one):** 🗖 Penicillin/gentamicin IV  🗖 Ceftriaxone once daily  🗖 Cefotaxime/cloxacillin IV  🗖 Penicillin only  🗖 Other: ___________________________________________________________  **Is patient HIV-exposed?**  🗖 Yes 🗖 No  Add high dose cotrimoxazole 🗖 Done 🗖 Not done  Refer for HIV testing 🗖 Done 🗖 Not done, HIV-infected 🗖 Not done | | | | |
| **NUTRITION STATUS/FEEDS**  **Is patient exclusively breast fed or on formular (choose one):**  🗖 Exclusively breast fed 🗖 Formula 🗖 Other:___________________________________________________  **Feeds/mode of feeding instituted on admission (choose one):**  🗖 EBM and BF 🗖 EBM via NGT 🗖 Formula by cup/spoon 🗖 Formula via NGT  🗖 Feeds withheld (state why):____________________________ 🗖 Other:________________________________  EBM: Expressed Breast Milk; BF: Breast feeding; NGT: Nasal gastric tube | | | | |
| **ASSESSMENTS**  **Blood culture:** 🗖 Done 🗖 Not done  Results: 🗖 *S. pneumoniae* 🗖 *H. influenza* 🗖 *S. aureus* 🗖 Other: _________________  **Complete blood count:** 🗖 Done 🗖 Not done  Results: WBC:________________ HB:___________________  Transfuse if severely anemic-HB <5g/dL  🗖 Done 🗖 Not done  **Chest x-ray:** 🗖 Done 🗖 Not done  Results: 🗖 Normal 🗖 Unilateral infiltrates 🗖 Bilateral infiltrates 🗖 Effusion 🗖 Cavitation  🗖 Hilar lymphadenopathy 🗖 Other: _____________________________ | | | | |
| **OXYGEN**  **Is room air saturation <92%**  🗖 Yes 🗖 No  Give supplemental oxygen by nasal cannula (start at 1L and increase to lowest level which attains oxygen saturation >92%) 🗖 Done 🗖 Not done | | | | |
| **SUPPORTIVE CARE**  **Is patient in shock (cold peripherals and capillary refill >3 seconds)?**  🗖 Yes 🗖 No  Correct the shock by administering a bolus of weight appropriate fluids 🗖 Done 🗖 Not done  Review the patient within one hour.  **Is patient febrile (temp>38.5^o^C)?**  🗖 Yes 🗖 No  Treat with paracetamol 15 mg/kg every 4 hours as needed 🗖 Done 🗖 Not done  **Is patient wheezing?**  🗖 Yes 🗖 No  Administer  salbutamol 🗖 Done 🗖 Not done  nebulizer Was there a  response to 🗖 Yes 🗖 No  salbutamol Administer 2 additional doses  of salbutamol nebulizations 🗖 Done 🗖 Not done  **Does patient have excessive nasal secretions?**  🗖 Yes 🗖 No  Perform nasopharyngeal suction 🗖 Done 🗖 Not done  **Is patient receiving maintenance fluids?** 🗖 Yes 🗖 No  Administer weight appropriate fluids by least invasive route possible  ___________ _____________  Initials Date | | | | |
|  | | **AT 48 HOURS** | **Ward number:** | |
| **ANTIBIOTICS**  **Does patient meet any criteria for treatment failure?** 🗖 Yes 🗖 No  If **YES**, choose one:  🗖 Add cloxacillin if not on cloxacillin  🗖 Switch to ceftriaxone or cefotaxime/cloxacillin if receiving penicillin and gentamicin  🗖 Switch to ciprofloxacin if receiving ceftriaxone/cefotaxime/cloxacillin  🗖 Switch to chloramphenicol instead of gentamicin  🗖 Switch to appropriate susceptible antibiotics if culture available: _______________________________________  ___________ ____________  Initials Date | | | | |

|  | | **ONGOING CARE** | | | | **Ward number:** | |
| --- | --- | --- | --- | --- | --- | --- | --- |
| **OXYGEN**  **Perform room air trial four times per day**  **Check the following:**   - Is room air saturation >92%? - Has chest indrawing resolved? - Is respiratory rate <70 breaths/min?   If **YES** to all, trial off oxygen and discontinue monitoring if room air saturation remains >92% for 15 minutes.  If **NO** to any, continue oxygen by nasal cannula  **or** if with signs of impending respiratory failure consider  mechanical ventilation and transfer to PICU. | | | | **SUPPORTIVE CARE**  **Check nasal cannula for occlusion four times per day.**  **If wheezing and responsive to salbutamol, continue salbutamol at minimum four times per day.**  **If febrile, continue paracetamol.**  **If secretions persist, continue suctioning.**  **Continue to provide maintenance fluids.**  **Ensure intake/administration of appropriate feeds.** | | | |
| ***Record results of room air trial in table below*** | | | | | | | |
|  | 1. **02H00** | | 1. **08H00** | | 1. **14H00** | | 1. **20H00** |
| **DATE: ____ / ____ / ____** |  | |  | |  | |  |
| Room air saturation |  | |  | |  | |  |
| Chest indrawing resolved? | 🗖 Yes 🗖 No | | 🗖 Yes 🗖 No | | 🗖 Yes 🗖 No | | 🗖 Yes 🗖 No |
| Respiratory rate |  | |  | |  | |  |
| Continue O_2_? | 🗖 Yes 🗖 No | | 🗖 Yes 🗖 No | | 🗖 Yes 🗖 No | | 🗖 Yes 🗖 No |
| Nasal cannula checked? | 🗖 Yes 🗖 No | | 🗖 Yes 🗖 No | | 🗖 Yes 🗖 No | | 🗖 Yes 🗖 No |
| Initials |  | |  | |  | |  |
| **DATE: ____ / ____ / ____** |  | |  | |  | |  |
| Room air saturation |  | |  | |  | |  |
| Chest indrawing resolved? | 🗖 Yes 🗖 No | | 🗖 Yes 🗖 No | | 🗖 Yes 🗖 No | | 🗖 Yes 🗖 No |
| Respiratory rate |  | |  | |  | |  |
| Continue O_2_? | 🗖 Yes 🗖 No | | 🗖 Yes 🗖 No | | 🗖 Yes 🗖 No | | 🗖 Yes 🗖 No |
| Nasal cannula checked? | 🗖 Yes 🗖 No | | 🗖 Yes 🗖 No | | 🗖 Yes 🗖 No | | 🗖 Yes 🗖 No |
| Initials |  | |  | |  | |  |
| **DATE: ____ / ____ / ____** |  | |  | |  | |  |
| Room air saturation |  | |  | |  | |  |
| Chest indrawing resolved? | 🗖 Yes 🗖 No | | 🗖 Yes 🗖 No | | 🗖 Yes 🗖 No | | 🗖 Yes 🗖 No |
| Respiratory rate |  | |  | |  | |  |
| Continue O_2_? | 🗖 Yes 🗖 No | | 🗖 Yes 🗖 No | | 🗖 Yes 🗖 No | | 🗖 Yes 🗖 No |
| Nasal cannula checked? | 🗖 Yes 🗖 No | | 🗖 Yes 🗖 No | | 🗖 Yes 🗖 No | | 🗖 Yes 🗖 No |
| Initials |  | |  | |  | |  |
| **DATE: ____ / ____ / ____** |  | |  | |  | |  |
| Room air saturation |  | |  | |  | |  |
| Chest indrawing resolved? | 🗖 Yes 🗖 No | | 🗖 Yes 🗖 No | | 🗖 Yes 🗖 No | | 🗖 Yes 🗖 No |
| Respiratory rate |  | |  | |  | |  |
| Continue O_2_? | 🗖 Yes 🗖 No | | 🗖 Yes 🗖 No | | 🗖 Yes 🗖 No | | 🗖 Yes 🗖 No |
| Nasal cannula checked? | 🗖 Yes 🗖 No | | 🗖 Yes 🗖 No | | 🗖 Yes 🗖 No | | 🗖 Yes 🗖 No |
| Initials |  | |  | |  | |  |
| **DATE: ____ / ____ / ____** |  | |  | |  | |  |
| Room air saturation |  | |  | |  | |  |
| Chest indrawing resolved? | 🗖 Yes 🗖 No | | 🗖 Yes 🗖 No | | 🗖 Yes 🗖 No | | 🗖 Yes 🗖 No |
| Respiratory rate |  | |  | |  | |  |
| Continue O_2_? | 🗖 Yes 🗖 No | | 🗖 Yes 🗖 No | | 🗖 Yes 🗖 No | | 🗖 Yes 🗖 No |
| Nasal cannula checked? | 🗖 Yes 🗖 No | | 🗖 Yes 🗖 No | | 🗖 Yes 🗖 No | | 🗖 Yes 🗖 No |
| Initials |  | |  | |  | |  |

***Continue on additional pages as necessary for the duration of hospital stay***

|  | **DISCHARGE** | **Ward number:** |
| --- | --- | --- |
| **Outcome of hospital stay:** 🗖 Discharged 🗖 Absconded 🗖 Died  **Does the patient meet each of the following discharge criteria:**  🗖 Able to feed without distress  🗖 No evidence of respiratory distress* If patient does not meet all 4 criteria  🗖 Room air saturation > 92% DO NOT DISCHARGE  🗖 Stable vital signs (temperature, pulse and blood pressure)  *retractions, nasal flaring, fast breathing, unable to tolerate medications | | |
| **ANTIBIOTICS**  **Choose oral regimen to complete 10 day course (choose one):**  🗖 Amoxicillin if receiving penicillin and gentamicin  🗖 Chloramphenicol PO if receiving chloramphenicol IV  🗖 Ciprofloxacin PO if receiving ciprofloxacin IV  🗖 Cefpodoxime PO if receiving ceftriaxone  🗖 Other susceptibility based antibiotic: _________________________________  **Is patient HIV exposed or infected?**  🗖 Yes 🗖 No  Continue cotrimoxazole 🗖 Done 🗖 Not done  Assess and refer for ART 🗖 Done 🗖 Not done  **Is patient Malnourished?**  🗖 Yes 🗖 No  Advise appropriately on infant nutrition 🗖 Done 🗖 Not done  Assess and consider discharging via A07 for nutritional rehabilitation | | |
| **DISCHARGE INSTRUCTIONS**  **Advise patient to return for review in 1 week**  🗖 Done 🗖 Not done  **Advise patient to return immediately if the child’s condition worsens or the child develops signs of respiratory distress.**  🗖 Done 🗖 Not done  **Describe signs of respiratory distress:** 🗖 Done 🗖 Not done   - Retractions - Nasal flaring - Fast breathing - Unable to tolerate medications   ­__________ ____________  Initials Date | | |
|  |  |  |
|  |  |  |
|  |  |  |
